# Methodology for Quantifying Volatile Compounds in a Liquid Mixture Using an Algorithm Combining B-Splines and Artificial Neural Networks to Process Responses of a Thermally Modulated Metal-Oxide Semiconductor Gas Sensor

**DOI:** 10.3390/s22228959

**Published:** 2022-11-19

**Authors:** Jolanta Wawrzyniak

**Affiliations:** Faculty of Food Science and Nutrition, Poznań University of Life Sciences, 60-624 Poznań, Poland; jolanta.wawrzyniak@up.poznan.pl

**Keywords:** thermally modulated gas sensors, metal-oxide semiconductor gas sensor, artificial neural network, machine learning, B-spline, quantitative determination, volatile component analysis

## Abstract

Metal oxide semiconductor (MOS) gas sensors have many advantages, but the main obstacle to their widespread use is the cross-sensitivity observed when using this type of detector to analyze gas mixtures. Thermal modulation of the heater integrated with a MOS gas sensor reduced this problem and is a promising solution for applications requiring the selective detection of volatile compounds. Nevertheless, the interpretation of the sensor output signals, which take the form of complex, unique patterns, is difficult and requires advanced signal processing techniques. The study focuses on the development of a methodology to measure and process the output signal of a thermally modulated MOS gas sensor based on a B-spline curve and artificial neural networks (ANNs), which enable the quantitative analysis of volatile components (ethanol and acetone) coexisting in mixtures. B-spline approximation applied in the first stage allowed for the extraction of relevant information from the gas sensor output voltage and reduced the size of the measurement dataset while maintaining the most vital features contained in it. Then, the determined parameters of the curve were used as the input vector for the ANN model based on the multilayer perceptron structure. The results show great usefulness of the combination of B-spline and ANN modeling techniques to improve response selectivity of a thermally modulated MOS gas sensor.

## 1. Introduction

Thanks to the rapid development of gas sensor technology in recent years these devices play an increasingly important role in many fields. Gas sensors designed so far have been widely used in environmental monitoring [1,2,3,4], medical assistance and diagnostics [5,6], and various types of industrial and agricultural production [7,8,9,10].

Among all the designed gas sensors, those based on metal-oxide semiconductors are of the greatest interest to researchers and manufacturers thanks to their high accuracy and sensitivity, short reaction times, long life, low power consumption and production costs [1,11,12,13]. They allow for the measurement of gases at levels from a dozen to several hundred ppm depending on the monitored volatile substance and the type of gas sensor [14,15]. The principle of operation of metal-oxide semiconductors is based on the coexistence of several physical processes and chemical reactions [16]. When voltage is applied to the sensor heater, the sensing material (semiconductors most often made of ZnO, Fe_3_O_4_, SnO_2_, CuO and NiO) heats up to a specific high temperature, which causes the electrons to pass from the forbidden to the allowed band. The released electrons partly participate in the current flow via a sensing layer (which is also powered with voltage) and partly are bound by the oxygen adsorbed on the semiconductor surface. The equilibrium established in this way is disturbed when a gas with reducing properties appears in the air. At a certain temperature, this gas undergoes a redox reaction, which leads to a change in the density of oxygen adsorbed on the detector. As a result, some of the electrons (previously attracted by oxygen) are released, enabling a more intense current flow, which may be measured as a diminution in sensor resistance. A comparison of the decreased resistance measured in an atmosphere containing reducing gases with the resistance measured in an atmosphere devoid of them can be used to determine their concentration.

Despite the many advantages of metal-oxide semiconductor gas sensors, the application of measuring modules containing only one sensor also has some drawbacks, as its response carries a limited amount of information. In the presence of a mixture of volatile compounds detectable by the abovementioned sensor, its response is the sum of the signals generated by individual substances and it is not possible to determine which part of the signal originates from which substance. This cross-sensitivity results in poor selectivity of the sensor, especially for gases such as ketones and alcohols, to which the sensor reacts with a similarly high response signal [17]. In consequence, quantification with the use of only one gas sensor is limited to the systems or atmosphere containing only one substance of a known type.

In view of the non-specific responses of gas sensors when exposed to a multi-component gaseous environment, the search for solutions increasing their selectivity is still the subject of many studies. Some inconvenience associated with the application of metal-oxide semiconductor gas sensors was overcome by including filters or catalysts (which are able to eliminate the influence of interfering gases) in the sensor design [18]. Research is ongoing to manufacture new gas-sensitive materials (e.g., nanomaterials) for the development of gas sensors [15,19,20]. The improvement of measurement selectivity has also been achieved by designing devices inspired by the olfactory system in form of matrixes containing groups of sensors with different characteristics (gas multi-sensor arrays), the so called electronic noses (e-noses) [16,21]. Although this technology is not without inherent flaws, it has found a wide range of applications, among others in recognizing crop diseases and insect pests [16], diagnosing the health of bee hives [14,22], estimating fungal infestation in rapeseed [23,24], estimating the source and quality of milk [7], assessing the quality of fermented foods and beverages [25], determining alcohol content [9,26] or evaluating the quality of raw material [27], etc.

The thermal modulation of metal-oxide semiconductor gas sensors proposed in recent years is another solution increasing their selectivity, and constitutes a promising approach for applications requiring the selective detection of individual components of a gas mixture [17,28]. It has been found that the varying input voltage applied to the gas sensor heater modulates the temperature of the sensor, which allows us to obtain additional information on the kinetics of the processes taking place on its surface [29,30]. As a result, the responses of a single thermally modulated metal-oxide semiconductor sensor can be treated as the action of several gas sensors of the same type operating under different temperature conditions; thus, reducing the problem of cross-sensitivity being a major obstacle to their widespread use [30,31].

As it was emphasized by Smulko et al. [18], the efficiency of gas detection can be enhanced not only by the development of sensor design, but also by the improvement of signal processing. In the literature, there are different approaches to represent the responses of MOS gas sensors. One of them is the use of the R_s_/R_0_ or R_0_/R_s_ ratio, which is applied especially in sensors with the heater powered by constant voltage [10,14,32,33]. The analyte concentration detected by MOS gas sensors can also be expressed as an output voltage measured in a voltage divider [9,26,27,34,35] and this method is often used in the case of sensors with thermal modulation of the heater [28,29,31]. It is worth emphasizing that both in the case of multi-sensor matrixes containing sensors with the heater powered by constant voltage and a single gas sensor with temperature modulation, output signals containing information on the type of volatile compound and its concentration take the form of unique, complex patterns; the interpretation of which requires the use of appropriate signal processing techniques. Since the method of analyzing output signals largely affects the accuracy of gas mixture composition identification, a number of approaches have been taken to improve the process of deciphering sensor responses. Principal Component Analysis (PCA) has often been used to distinguish gas sensor response patterns and to classify substances contained in an analyzed gas mixture [28,30,35]. In other cases, the output signal recognition has been carried out by PCA or linear discriminant analysis (LDA) combined with such methods as the k-nearest neighbor method (KNN), logistic regression (LR), support vector machine (SVM) or random forest (RF) algorithm [7,29,36,37]. Partial Least Squares (PLS) regression is another method that has been proposed for classifying multi-sensor responses when identifying the infestation rate of *Varroa destructor*, a parasite attacking honeybee colonies [14]. In recent years, signal processing combined with non-linear calibration and machine learning methods has been gaining in importance and is seen as a prospect to improve selectivity and detection accuracy under real atmospheric conditions [9,11,26,38,39].

As shown in the literature, research on modern, highly-efficient, intelligent gas sensors characterized by increased selectivity is carried out intensively. Nevertheless, there is still a great demand for research developing new measuring and signal processing methods to provide better exploration of gas sensor output patterns. In this study, the hypothesis was assumed that it is possible to elaborate: (a) a methodology of measurement based on a single thermally modulated metal-oxide semiconductor gas sensor, and (b) an algorithm based on the B-spline curve and artificial neural networks (ANNs) to selectively process and interpret output signals of this sensor, which can be used to estimate concentrations of volatile components contained in liquid mixtures.

## 2. Materials and Methods

### 2.1. Gas Sensor and Measuring System

**Figure 1:**
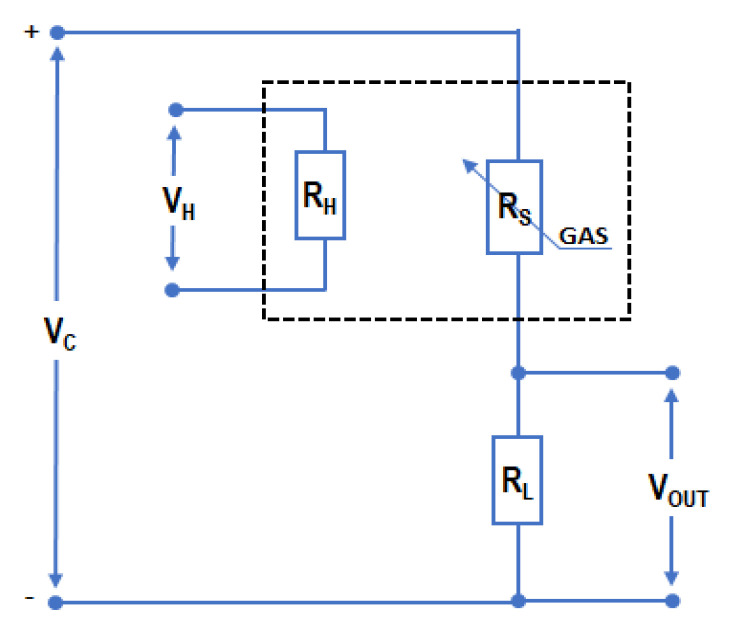
The scheme of the measuring system of the TGS2610-C sensor, where R_S_—sensor resistance; R_L_—resistance of an auxiliary (load) resistor; V_C_ and V_H_—voltage of sensor circuit and micro-heater circuit; V_OUT_—voltage related to sensor resistance R_S_ measured in a voltage divider system using an auxiliary resistor R_L_.

In the study, all measurements were carried out using a single metal-oxide semiconductor TGS2610-C gas sensor manufactured by Figaro Engineering Inc. Osaka, Japan. The sensing element of the applied gas sensor chip consists of a thin tin-oxide semiconductor film formed on an alumina underlay integrated with a microheater in the form of a printed layer of RuO_2_ material. Figure 1 presents the scheme of the measuring circuit of the TGS2610-C sensor. The variation of sensor resistance R_S_ is measured in a voltage divider system using an auxiliary (load) resistor R_L_ and expressed via the value of output signal voltage (V_OUT_). 

In a typical manufacturer-recommended configuration, both the voltage powering the sensor circuit (V_C_) and the voltage powering the micro-heater circuit (V_H_) should have a constant value of 5.0 V. In this study, the circuit prepared according to the conventional methodology (Figure 1) was applied, but to increase sensor selectivity, its heater was subjected to thermal modulation. For this purpose, the heater circuit was powered with voltage (V_H_) linearly increasing at the rate of 0.5 V∙min^−1^ in the range between 0 to 5 V. The temperature variation influenced the intensity of electron transition from the forbidden to the allowed band and also the intensity of the adsorption and redox reactions on the surface of the sensing material, thus affecting the sensor resistance Rs, the changes of which were registered as the output signal across the R_L_ in a voltage divider system. Measurement of the output voltage V_OUT_ as a function of the heater voltage V_H_ made it possible to plot the characteristics containing more detailed information about the composition of the tested mixtures.

### 2.2. Preparation of Samples for Analysis

The aqueous mixtures of two volatile organic compounds, ethanol and acetone, were examined in the study. The tested solutions contained a combination of thirteen different concentrations of each of the aforementioned analytes ranging from 0.0078% to 0.5%. Since the solution was prepared by the serial dilution method, it was convenient to handle concentrations in the form of a power of two, i.e., from 2^−7^% to 2^−1^% (corresponding to analyte concentration of 78, 110, 156, 221, 312, 442, 625, 884, 1250, 1768, 2500, 3536, 5000 ppm). Ethanol (≥99.8%) and acetone (≥99.9%) were purchased from POCH. The solutions were prepared on the basis of deionized water

### 2.3. Experiment Design

The selected volatile components were analyzed in a 2 L chamber equipped with a fan and the gas sensor measurement setup. Samples of 50 mL of solutions containing specified concentrations of acetone and ethanol were placed inside the chamber, and after waiting 5 min in order to achieve a thermodynamic balance between the sample and the gas phase, the measurement was performed. Sensor response patterns for the analyzed gas mixture were recorded as the relationship between the voltage of the micro-heater (V_H_) and the voltage of the output signal (V_OUT_). Measurements were recorded for 13 different levels of each determined analyte, i.e., ethanol and acetone, in liquid samples (in total 13 × 13 = 169 tests were carried out in triplicate, each of which contained 501 measurement points). Before each measurement, the chamber was ventilated with fresh air.

### 2.4. Gas Sensor Output Signal Processing

**Figure 2:**
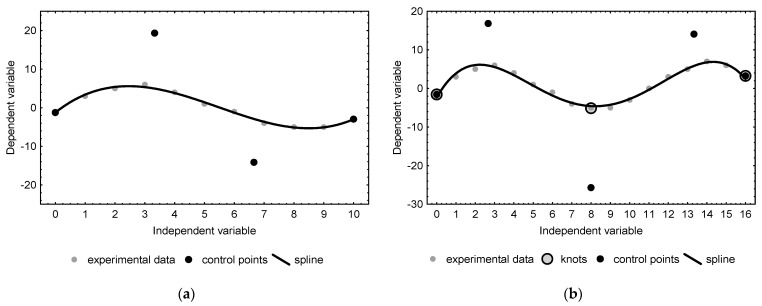
Diagrams showing the distribution of knots and control points when approximating data using the B-spline method for (**a**) a single polynomial reflecting a data set with a run close to the third-order polynomial (4 auxiliary points), and (**b**) a B-spline consisting of two polynomials reflecting a data set with a run close to the fourth order polynomial (5 control points).

The response patterns of the thermally modulated gas sensor registered in the experiments contained information on the contents of the analytes in tested solutions; hence, in further tests, they were intended to be used as input data to design a neural network model estimating the concentrations of ethanol and acetone (ANN_E-A_) in liquid mixtures. Since 501 output voltage points were recorded in each experiment corresponding to the defined analyte levels, the use of such a large set of raw data as an input vector for ANNs could lead to an excessive increase in computational complexity. With this in mind, an attempt was made to reduce the size of the recorded data set while maintaining the maximum possible amount of information contained in it. For this purpose, the data set was compressed with the use of a B-spline, which is a function described piecewise by polynomials and is able to efficiently approximate complex dependencies. In spline approximation methodology, data reflecting a simple relationship can be described by a single polynomial, defined by a set of the so-called control points, the number of which must be greater than its degree by one, i.e., two for a linear, three for a quadratic, four for a cubic function, etc. Out of the control points, two coincide with the first and last point in the dataset, while the others, located between them, but usually not coinciding with the points belonging to the dataset, modulate the curve so as to minimize the approximation error (Figure 2a). Formally a polynomial approximating data can be of any degree, but in practice usually not higher than tertiary polynomials are used. More complex datasets, which would require the use of higher-degree polynomials are approximated by a piecewise function (Figure 2b).

When designing a B-spline, some of the points from the data set (always including the first and the last) are selected to act as knots dividing the data set into intervals, which in the next step are approximated with independent polynomials. A characteristic feature of the spline curve is that it always passes through knots (and hence by the measurement points that have been selected to act as knots). Its course inside each interval is shaped by control points, which are designated under the following assumptions: (1) the first and last control points coincide with the first and last knots (and at the same time coincide with the first and last point from the data set); (2) the abscissa of the second control point is between the abscissa of the first and the second knot, similarly the abscissa of the last but one control point is between the abscissa of the last but one and last knot; (3) the abscissa of the control points from the third to the last but two is the same as the abscissa of the knots from the second to the last but one. The principle of approximation with the use of a B-spline is to arrange knots and control points in such a way that, using their relatively small number, the created curve effectively approximates the data set. The methodology of calculating intermediate values of the curve is widely described in the literature and implemented in popular programming languages, and in the broadest sense it consists in “attracting” the curve by knots and control points [40].

In this study, each experimental series of data was fitted with B-spline curves whose parameters were determined using the LSQ Univariate Spline method from scipy.interpolate module in the Python Programming Language. During the development of the B-spline curves, tests were carried out to reduce (taking into account the goodness of data fitting) the number of knots and control points making up the curves. The modeling process began with designing a B-spline based on 51 knots (one knot coinciding with the starting point in the data set, 49 intermediate knots evenly spaced at every 0.1 V and one knot coinciding with the end point in the data set). Then, the knots, the deletion of which resulted in the smallest increase in the curve-fitting error, were gradually (as long as their number reached six) removed. For each experimental waveform (recorded for different configurations of analyte concentrations), the control points were read and then the input vectors for the neural network were created on their basis.

### 2.5. Development of an Artificial Neural Network Model

Artificial neural networks constitute a modeling technique that in many ways resembles the structure and action of the human brain, and which work well in describing non-linear phenomena [41,42]. The ANN architectures are optimized in the learning process and consist of basic elements—neurons (nodes) connected by synaptic weights into a multi-layer structure. The flexible structure and capability of ANNs for efficient parallel information processing without prior requirements and assessments determine high usefulness of artificial neural networks in describing complex relationships.

In this study, an ANNmodel for quantitative determination of analyte concentrations in liquid mixtures was developed based on the multilayer perceptron (MLP) using Statistica 13.3 software (StatSoft, Tulsa, OK, USA). The constructed model was a feedforward neural network, in which the entered signal passes through its multilayer structure from the input layer through hidden layer(s) to the output layer. The ANN model was built based on the results of 169 experiments (each comprising 501 measurements) that were randomly divided into the learning, test and validation data sets at the ratio 70, 15 and 15%, respectively. The optimization of the model structure was carried out based on the learning and test data sets, the first of which was used in the ANN construction, while the latter was used to verify its operation during the machine learning process. The accuracy and generalization capability of developed an ANN model were assessed based on the validation data set that was not included in the network design process. The input signal of each tested ANN constituted the vector composed of eight control points calculated during the spline curve designing for each experimental system, whilst the output signals were the logarithm based upon two of the ethanol and acetone concentrations in the analyzed solutions. During the optimization of the model structure, networks with a single hidden layer containing from 2 to 9 neurons were examined. For each tested topology, the influence of the type of activation function (linear (Lin), logistic (Log), exponential (Exp) and hyperbolic tangent (Tanh) in the neurons of the hidden layer was additionally investigated. Since previous studies have shown that in networks operating in the regression mode the output neurons with a linear function worked best [43,44], this type of function was used in the designed model. The parameters of the ANN were estimated using the Broyden–Fletcher–Goldfarb–Shanno (BFGS) learning algorithm. The sum of squares was the error function while designing the network. At each stage of model construction, the predictive quality of the examined network was assessed on the basis of the learning, test and validation error. 

### 2.6. Statistical Assessment of ANN_E-A_ Model Performance

The Statistica 13.3 software (StatSoft, Tulsa, OK, USA) was used to assess the capability of the developed ANN_E-A_ model. The goodness of fitting the model response to the experimental points was evaluated using a determination coefficient (R^2^). The model accuracy was also appraised by the mean absolute error (MAE) and the root mean square error (RMSE) expressed with the following expressions:

(1) $$\mathrm{MAE}=\frac{1}{n}\cdot \sum \left|C_{E}-C_{M}\right|$$

(2) $$\mathrm{RMSE}=\sqrt{\frac{\sum {\left(C_{E}-C_{M}\right)}^{2}}{n}}$$

where: *n* is the number of experimental points; C_E_ is the experimental analyte concentration %; and C_M_ is the estimated analyte concentration %. All the tests and calculations were performed at the significance level of α = 0.05.

## 3. Results and Discussion

### 3.1. Dynamic Response Signal of Gas Components

**Figure 3:**
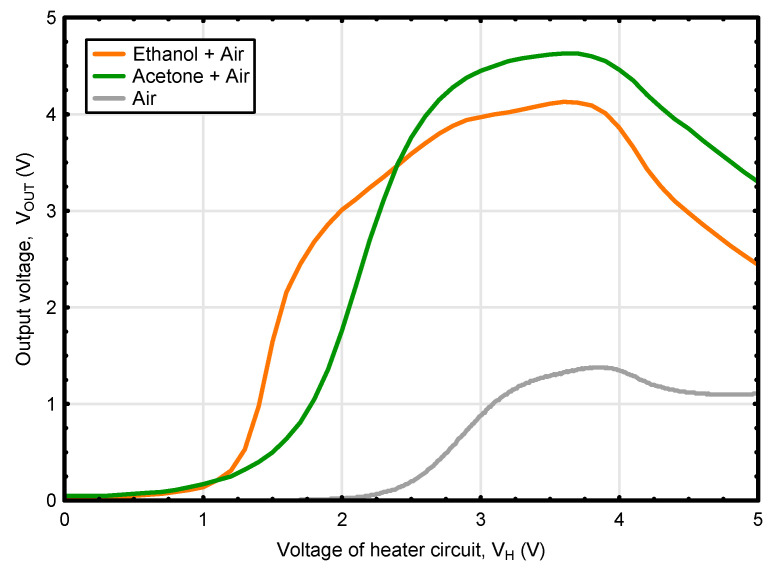
V_OUT_ waveforms as a function of linearly increasing voltage (at rates of 0.5 V·min^−1^) of the micro-heater of the TGS2610-C gas sensor obtained individually for fresh air and for solutions containing maximal tested concentrations (0.5%) of individual analytes: ethanol or acetone.

In this study, the procedure for determining the concentrations of ethanol and acetone in liquid mixtures was elaborated based on the dynamic responses of the thermally modulated gas sensor exposed to the atmosphere being in the state of thermodynamic equilibrium with the analyzed solutions. The considerations of the abovementioned procedure started with the analysis of the waveforms of output voltage V_OUT_ recorded as a function of the sensor micro-heater voltage V_H_ for fresh air and for the solutions containing the maximum tested concentrations (0.5%) of the individual analytes (Figure 3). It was easy to notice that the obtained curves vary significantly in their course. The changes in V_OUT_ for acetone started to rise later, but its increase was more intense and reached a higher maximum level (4.65 V) than for ethanol (4.13 V). Durán et al. [35] observed the comparable response patterns of V_OUT_ for ethanol measured in wine samples using gas sensors, to which the modulation method was applied. In turn, a similar waveform of output voltage for acetone, examined as a single gas component with the use of a tin-oxide-based sensor (SP3-AQ2, FIS Inc., Hyogo, Japan), was recorded by Hossein-Babaei and Amir Amini [28].

**Figure 4:**
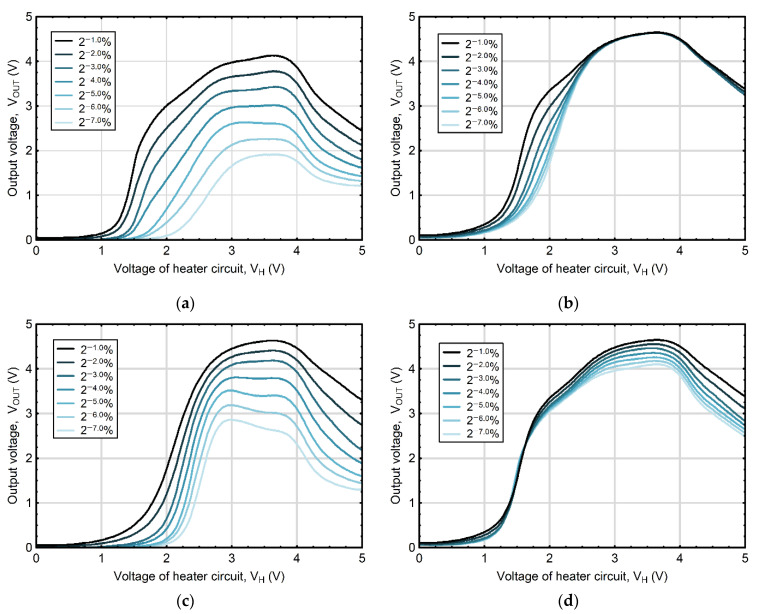
The voltage output signal of the TGS2610-C gas sensor with temperature modulation for different concentrations of (**a**) ethanol alone, (**b**) ethanol at the maximum analyzed level of acetone (0.5%), (**c**) acetone alone, and (**d**) acetone at the maximum analyzed level of ethanol (0.5%). For transparency and legibility, the graphs show the voltage output signal of the thermally modulated gas sensor (TGS2610-C) for every second combination of the applied concentrations of tested analytes.

As it was emphasized by Bora and Sarma [31], the characteristic response waveform is a signature of a particular gas. Nevertheless, the research conducted so far has focused on the identification of gaseous components in the mixture, but without their quantitative determination [28,29,30,38]. The fact that the curves obtained for acetone and ethanol showed different courses (Figure 3), led to the assumption that it would be possible to establish their concentrations in mixtures. To verify this hypothesis, the atmosphere being in equilibrium with liquid solutions containing different concentrations of acetone and ethanol (in the range 0.0078–0.5%) was examined with a single metal-oxide semiconductor TGS2610-C gas sensor with heater thermal modulation. Figure 4a–d depicts the course of the changes in the voltage output signal of the used gas sensor recorded for different concentrations of tested analytes in the liquid mixtures, while completely devoid or maintaining the maximum level of the accompanying compound. 

The obtained results indicated that the output signals of the sensor V_OUT_ recorded for solutions containing different concentrations of individual analytes have repeatable patterns that grow with an increasing content of the analyzed compounds. However, in the presence of the accompanying analyte, the response patterns were of a different nature depending on the level of this concomitant substance. Figure 4b,d depicts that in the case of solutions, in which the various concentrations of individual analytes occurred with a maximum concentration of the second analyzed substance, an increase in the V_OUT_ value was observed only on some sections of the curves. The shapes of the waveforms and changes in V_OUT_ values recorded for varying concentrations of the analyte indicates that the obtained output signals contain information on the type and concentration of the analyzed substances in the tested solutions. However, due to the complexity of the relationships, their direct quantitative interpretation is not feasible and requires advanced processing techniques.

### 3.2. Qualitative and Quantitative Analyses

The complexity of the experimental results shows the importance of proper selection of the technique used to process data. The solution for this problem was provided by the use of ANNs, which thanks to their computational capabilities, flexibility and ease of use, are the preferred tool in many applications, especially those where a description of complex nonlinear phenomena is required [45,46]. In the case of the data recorded in this study, some difficulties in the ANN application could be caused by the relatively large size of the input vector (501 points obtained for each experimental system as a response of the heater voltage modulated in the range from 0 to 5 V with a resolution of 0.01 V). Therefore, before data modeling with the ANN, it was necessary to reduce the size of the input data vector while maintaining all its essential features.

**Figure 5:**
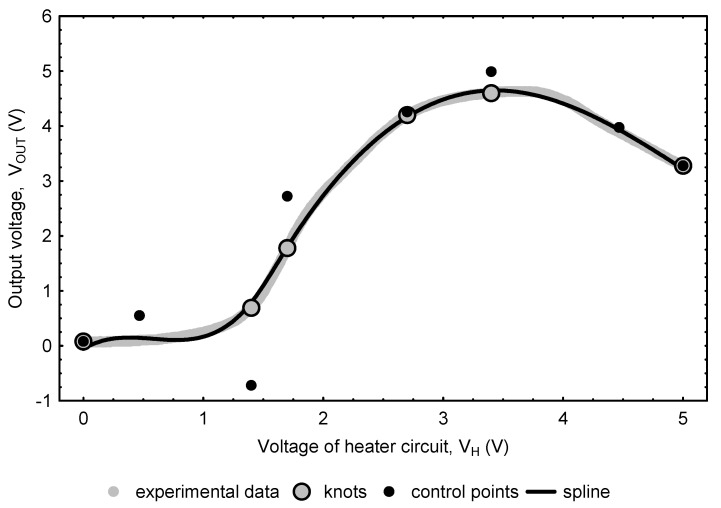
An example waveform of V_OUT_ as a function of linearly increasing the micro-heater voltage of the TGS2610-C gas sensor obtained for the aqueous mixture of ethanol and acetone.

Spline curves, similarly as Bezier curves, are often used for shape modeling in professional graphic and CAD software [40]. Taking advantage of this technique, an attempt was made to “compress” the data obtained in the measurements by approximating them with the B-spline curves described by a specific set of knots and control points. In the study, curves based on a different number of nodes, starting with 51, were tested in terms of the quality of the experimental data approximation. Multiple repetition of the knot elimination procedure that was applied during the B-spline design process allowed us to decrease the number of knots to six (which corresponds to eight control points). Figure 5 shows a spline curve approximating the measurement data of a single exemplary data set based on selected knots and control points. 

The obtained results showed that the removal of the least important knots (located mainly in areas with low variability) made it possible to obtain a significant “compression” of the sensor output signal while maintaining a good fit of the designed B-spline curve to the experimental data. The approximation of the waveforms recorded during the measurements with the use of the described technique made it possible to transform them into a new smaller vector, which while maintaining information contained in the parent data, was composed of the eight coordinates of control points shaping the B-spline curve.

### 3.3. Artificial Neural Network Modeling

**Figure 6:**
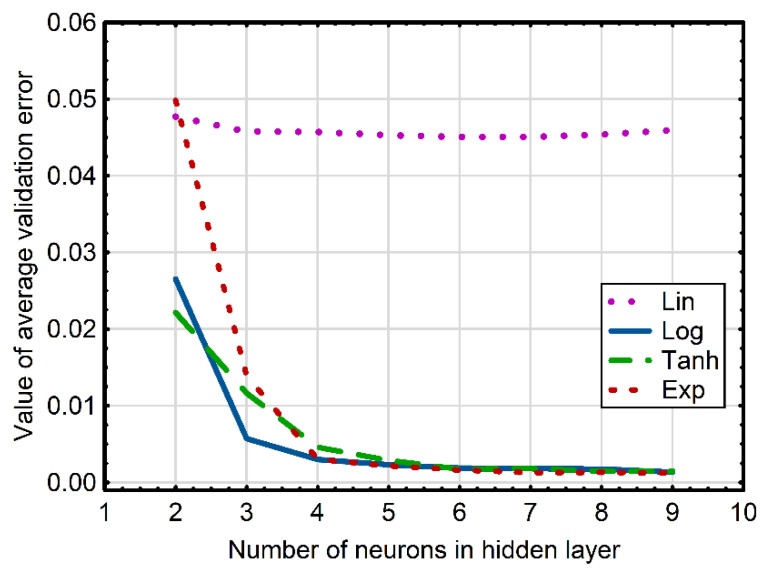
The impact of the number of nodes and the type of activation function (linear (Lin), logistic (Log), exponential (Exp) and hyperbolic tangent (Tanh)) in neurons of the hidden layer on the average value of the learning, test and validation errors of the examined networks describing levels of acetone and ethanol in analyzed mixtures.

Since previous studies have shown that the single hidden layer MLP performs well in mapping non-linear relationships [47,48,49,50], this structure was used in this study to construct the ANN model quantifying acetone and ethanol levels in the tested mixtures (ANN_E-A_). Assuming that the designed B-spline curves encode the information obtained from the measurement data, the input vector of the neural network was constructed based on control points shaping these curves. The designed MLP-based model comprised eight input neurons corresponding to the number of spline control points, and two output neurons corresponding to ethanol and acetone concentrations. When building the ANN_E-A_ model, much attention has been paid to determine the structure of the hidden layer. As there are no universal and widely accepted rules for designing the architecture of neural networks [51], in this study, it was designed through the trial and error method. In consequence, various network topologies were tested, which differed in the number of neurons of the hidden layer and the type of their activation function. For each size of the hidden layer and for the type of activation function in neurons of the hidden layer one thousand networks were generated. In total, 32,000 MLP networks (eight hidden layer structures × four types of the transfer function in neurons of the hidden layer × one thousand networks for each topology) were tested. The results of this analysis on the impact of the number of nodes and the type of activation function in the neurons of the hidden layer on the average value (out of 1000 networks designed for each topology) of the validation errors for the examined networks are shown in Figure 6.

It is evident that the networks, in which the neurons of the hidden layer were equipped with a linear activation function, are characterized by a high validation error regardless of the number of neurons in this layer (the average validation error was above 0.045). These results indicate limited usefulness of these groups of networks in modeling ethanol and acetone contents in the tested liquid mixtures. Among the studied network topologies, networks with neurons of the hidden layer comprising a non-linear activation function, i.e., a hyperbolic tangent, exponential and logistic function, proved to be more effective in modeling the concentration of tested analytes. The expansion of the hidden layer in these networks from 2 to 4 neurons resulted in an intensive decrease in the value of the average validation error. A further increase in the size of the hidden layer did not significantly improve its value, which after the initial downward trend stabilized at a similar level (below 0.005) for all structures containing four or more nodes regardless of the type of activation function in neurons of the hidden layer. When optimizing the network topology, it should be remembered that a too small structure usually results in poor mapping of dependencies in the modeled systems and high validation error [52]. As the complexity of the network increases, there is usually a decrease in learning, test and validation errors. Nevertheless, it is worth emphasizing that an overly extended structure of the network can lead to network overfitting and the memorization of dependencies between the input and output data, which in turn, would result in the loss of the network’s ability to generalize its existing dependencies on new data [52,53]. As model structure should be characterized by the simplest possible structure while maintaining high-approximation quality and generalization ability, in the study, the ANN model was searched for in the group of networks containing four neurons in the hidden layer. The low values of the validation error observed for this group of ANN structures and the small size of the network ensured that they would not be overtrained and would perform well on the new data. Among these topologies, the best predictive quality and the ability to generalize data not participating in the construction of the model (confirmed by the lowest validation error) were noted for an MLP network, in which four neurons of the hidden layer were equipped with a hyperbolic tangent activation function. This artificial neural network was selected for a model quantifying acetone and ethanol levels in the analyzed solutions. Table 1 presents metrics of the ANN adopted for the model estimating ethanol and acetone concentrations in liquid mixtures. 

**Figure 7:**
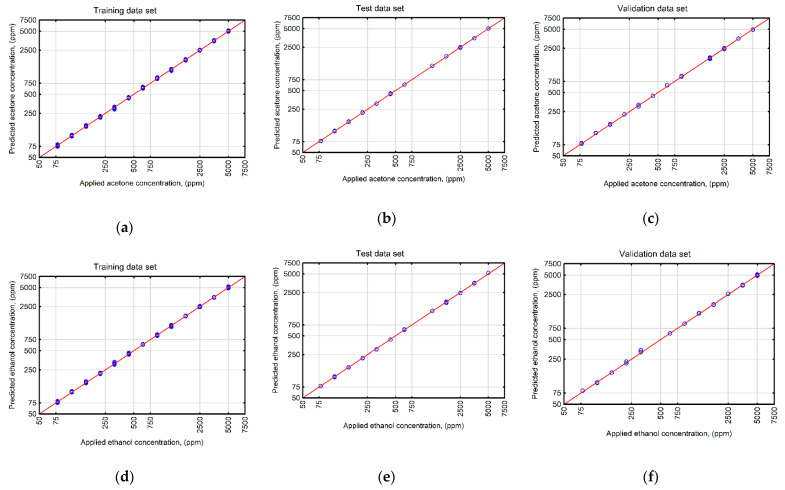
A comparison of analyte concentrations (ethanol and acetone) applied in experiments and estimated with the ANN_E-A_ model for (**a**) learning, (**b**) test, (**c**) validation data sets recorded for acetone and (**d**) learning, (**e**) test, (**f**) validation data sets recorded for ethanol.

The values of the learning, test and validation errors obtained during the network optimization process indicated that the topology of the selected network constituted a good compromise between the simplicity of structure and predictive efficiency. The correlation analysis between the experimental data and outcomes returned by the network confirmed a high efficiency of the developed model. Figure 7 shows good agreement between the model predictions and the tested analyte concentrations.

The values of statistical indicators calculated during the evaluation of the developed neural network model performance also showed its high reliability (Table 2). 

The values of the coefficient of determination (R^2^) computed for the learning, test and validation data sets showed high accuracy of the model response. A high value of the coefficient of determination obtained for the validation data set not included in network topology optimization (R^2^ = 0.9997−0.9998) also indicated that the designed network was not over-trained and showed good predictive efficiency and a high generalization capability on new data. The high degree of closeness of the model outcomes with the concentrations of the tested analytes in the liquid mixtures used in the experiments was also confirmed by low values of the root mean square error (RMSE), describing the mean deviation between the model estimation and the experimental points and the mean absolute error (MAE), being a measure of the average absolute deviation between the abovementioned data sets. The results obtained in this study show a high degree of usefulness for the combination of spline curve approximation and ANNs in improving the response selectivity of a thermally modulated metal oxide semiconductor gas sensor. The presented approach is an extension of the methodology used so far to analyze gases in mixtures, as it provides an experimental idea and a method of increasing the selectivity of dynamic response signal interpretation. Thus, further research is planned on the applicability of the described methodology for solutions containing more than two volatile substances and comprising both volatile and non-volatile compounds.

## 4. Conclusions 

Responses of thermal modulated gas sensors can be treated as a source of data on the composition of the analyzed gas mixture, but their quantitative interpretation is a difficult challenge. The paper presents the methodology of measuring the concentration of volatile components in a mixture using a thermally modulated metal oxide semiconductor gas sensor and proposes an algorithm that significantly extends the scope of exploration of the data recorded for this type of detector. In the applied approach, the use of the spline approximation technique to process the measurement data allowed us to extract the basic “knowledge” from complex nonlinear gas sensor response patterns and transform them into a reduced input vector for ANNs. The designed neural network model estimated the concentrations of the tested analytes (acetone and ethanol) contained in the liquid solutions with satisfactory accuracy; thus, indicating the usefulness of this technique in the interpretation of gas sensor response patterns. The obtained results suggest that the use of a single thermally modulated gas sensor in conjunction with an algorithm based on the B-spline curve and ANNs to register and interpret output signals extends the area of their application. Moreover, it is worth emphasizing that the proposed technique (consisting in compressing a data vector with a spline and then using it as an input vector for an ANN) is universal and can also be used in the analysis of other types of spectral data.

## Figures and Tables

**Table 1:** Basic information on the architecture and error values for the learning, test and validation process of the MLP neural network selected as a model estimating ethanol and acetone concentrations in liquid mixtures based on the response of a single thermally modulated metal-oxide semiconductor gas sensor.

Network Parameters	Artificial Neural Network MLP 8-4-2
Number of observation points (total)	169
Learning	119
Test	25
Validation	25
Activation functions in hidden layer	Tanh
Activation functions in output layer	Lin
Learning error	0.00076
Test error	0.00050
Validation error	0.00127
Learning accuracy	0.9999
Test accuracy	0.9999
Validation accuracy	0.9998

**Table 2:** Values of indicators used to evaluate the performance of the ANN_E-A_ model to predict the contents of ethanol and acetone in a liquid mixture.

Statistical Index	Model ANN_E-A_	
	Ethanol	Acetone
Coefficient of determination (R^2^)	0.9994	0.9997
Root mean square error (RMSE)	0.000014	0.000007
Mean absolute error (MAE)	0.001832	0.001570

## Data Availability

All data are available from the corresponding author upon request.
